# (+)-Usnic Acid Inhibits Migration of c-KIT Positive Cells in Human Colorectal Cancer

**DOI:** 10.1155/2018/5149436

**Published:** 2018-09-12

**Authors:** Wei Wu, Bing Hou, Changli Tang, Fucheng Liu, Jie Yang, Tao Pan, Ke Si, Deyun Lu, Xiaoxiang Wang, Jing Wang, Xing Xiong, Ji Liu, Chunguang Xie

**Affiliations:** ^1^Department of Gastroenterology, Integrated Traditional Chinese Medicine and Western Medicine Hospital Affiliated to Chengdu University of Traditional Chinese Medicine/Chengdu First People's Hospital, Chengdu 610041, China; ^2^School of Clinical Medicine, Chengdu University of Traditional Chinese Medicine, Chengdu 610075, China; ^3^Chengdu Easton Biopharmaceuticals Ltd., Chengdu 611731, China; ^4^Department of Biochemistry and Molecular Biology, West China School of Basic Medical Science and Forensic Medicine, Sichuan University, Chengdu 610041, China; ^5^Remeadjohn Technology Co., Ltd., Chengdu 610044, China; ^6^Pharmacy Department, Xichang People's Hospital, Xichang 615000, China

## Abstract

Inhibition of tumor cell migration is a treatment strategy for patients with colorectal cancer (CRC). SCF-dependent activation of c-KIT is responsible for migration of c-KIT positive [c-KIT(+)] cells of CRC. Drug resistance to Imatinib Mesylate (c-KIT inhibitor) has emerged. Inhibition of mTOR can induce autophagic degradation of c-KIT. (+)-usnic acid [(+)-UA], isolated from lichens, has two major functions including induction of proton shuttle and targeting inhibition of mTOR. To reduce hepatotoxicity, the treatment concentration of (+)-UA should be lower than 10 *μ*M. HCT116 cells and LS174 cells were employed to investigate the inhibiting effect of (+)-UA (<10 *μ*M) on SCF-mediated migration of c-KIT(+) CRC cells. HCT116 cells were employed to investigate the molecular mechanisms. The results indicated that firstly, 8 *μ*M (+)-UA decreased ATP content via uncoupling; secondly, 8 *μ*M (+)-UA induced mTOR inhibition, thereby mediated activation suppression of PKC-A, and induced the autophagy of the completed autophagic flux that resulted in the autophagic degradation and transcriptional inhibition of c-KIT and the increase in LDH release; ultimately, 8 *μ*M (+)-UA inhibited SCF-mediated migration of CRC c-KIT(+) cells. Taken together, 8 *μ*M could be determined as the effective concentration for (+)-UA to inhibit SCF-mediated migration of CRC c-KIT(+) cells.

## 1. Introduction

Colorectal cancer (CRC) is characterized by the fourth most familiar cause of cancer-related death in the world [1]. Metastasis of tumor cells is the predominant cause of mortality in patients with CRC [2]. Accordingly, inhibition of tumor cells migration is a treatment strategy for reducing the mortality of CRC patients [3].

Mast/stem cell growth factor receptor Kit (c-KIT), a tyrosine-protein kinase-type receptor for cytokine Kit ligand (SCF), plays a significant role in the regulation of cells migration [4]. A previous study has reported that (1) 50% of human CRC samples exhibited at least low expressions of c-KIT; (2) and in human CRC, c-KIT was specifically expressed in one subset of CRC cells [5]. SCF-dependent activation of c-KIT will promote migration of c-KIT(+) CRC cells [6]. Therefore, inhibition of c-KIT activity may attenuate SCF-mediated migration of c-KIT(+) CRC cells [7]. As a tyrosine kinase inhibitor (TKI), Imatinib Mesylate can bind to tyrosine kinases pocket of c-KIT and competitively inhibit ATP binding, thereby effectively maintaining an inactive conformation of c-KIT [8]. However, TKI resistance will probably be triggered by c-KIT mutation or the long-term use of TKI [9]. A recent research has shown that serine/threonine protein kinase mTOR (mTOR) inhibitor can effectively induce autophagic degradation of c-KIT in gastrointestinal stromal tumor cells [10]. As mentioned earlier, c-KIT is also expressed in a subset of human CRC cells [5], which endows mTOR inhibitor with the possibility of inducing autophagic degradation of c-KIT in this subset of human CRC cells. Furthermore, in autophagy process, the efficient protein quality control requires upregulation of SUMO-conjugating enzyme UBC9 (UBC9) [11]. Upregulated UBC9-mediated increase in sumoylation of transcription factor AP-2-alpha (TFAP2A) will negatively regulate AP-2 transcriptional activation [12], which will mediate transcriptional inhibition of c-KIT [13]. Consequently, inhibition of mTOR may probably suppress SCF-mediated migration of c-KIT(+) CRC cells via inducing the autophagic degradation and transcriptional inhibition of c-KIT. In addition, a previous study has indicated that mTOR inhibitors can inhibit cell motility via activity inhibition of protein kinase C alpha type (PKC-A) induced by inhibition of mTOR complex 2 (mTORC2) [14]. Rapamycin, a classic inhibitor of mTOR, can effectively inhibit mTOR complex 1 (mTORC1) [15]. But the drug resistance to rapamycin will be triggered by mTOR-p70s6k-irs1 negative feedback [16]. It is important to find the novel mTOR inhibitors.

(+)-UA (GAS: 7562-61-0) is isolated from lichens that is widely used as an antimicrobial agent in Traditional Chinese Medicine [17]. In fact, (+)-UA has two major pharmacological functions including targeting inhibition of mTOR and induction of proton shuttle [18, 19]. Firstly, (+)-UA displays an optimal binding posture at mTOR kinase pocket to effectively inhibit the functions of mTORC1/2 [18], which may induce the autophagic degradation and transcriptional inhibition of c-KIT as previously described. Secondly, lipophilic- and weakly acidic- (+)-UA can effectively mediate mitochondrial proton shuttle (uncoupling) that results in ATP content decrease [19], which would directly attenuate cell motility [20]. Thirdly, as the end results of targeting inhibition of mTOR and induction of proton shuttle, the treatments of different cancer cell lines with 12.5-100 *μ*M of (+)-UA can effectively mediate autophagic death or apoptosis in a time and dose dependent manner [18, 19, 21, 22]. Hence, there is a functional synergy between targeting inhibition of mTOR and induction of proton shuttle that probably endows (+)-UA with the ability to inhibit SCF-mediated migration of c-KIT(+) CRC cells. Meanwhile, (+)-UA would also mediate hepatocyte toxicity in a dose and time dependent manner [23]. Further study found that, for (+)-UA-treated primary cultured rat hepatocytes for 24 h, 10 *μ*M seems to be the concentration threshold for hepatocytes to overcome (+)-UA mediated hepatocyte toxicity by increasing oxidative phosphorylation [24]. Therefore, in order to reduce the adverse reaction of liver injury, the treatment concentration of (+)-UA on cells should be lower than 10 *μ*M. Nevertheless, there is still no research that involves whether or not the treatment of c-KIT(+) CRC cells with (+)-UA (<10 *μ*M) can inhibit SCF-mediated cells migration.

Notably, cell viability assays had indicated that the IC50 values for (+)-UA against human CRC HCT116 cells and LS174 cells were 17.7 *μ*M and 15.66 *μ*M, respectively [25, 26]. Furthermore, c-KIT is expressed in HCT116 cell line and LS174 cell line, respectively [5, 27]. In this study, HCT116 cells and LS174 cells would be employed to investigate and confirm the inhibiting effect of (+)-UA (<10 *μ*M) on SCF-mediated migration of c-KIT(+) CRC cells. And because HCT116 cell line is a suitable transfection host, HCT116 cells would be employed to investigate the molecular mechanisms for (+)-UA (<10 *μ*M) to inhibit SCF-mediated migration of c-KIT(+) CRC cells.

## 2. Materials and Methods

### 2.1. Chemicals and Reagents

(+)-*μ*snic acid, Dulbecco's Modified Eagle's Medium (DMEM), William's E medium, thiazolyl blue tetrazolium bromide (MTT), Kit ligand (SCF), Go-6976, dimethylsulfoxide (DMSO), and fetal bovine serum (FBS) were purchased from Sigma-Aldrich (St Louis, MO). Antibiotic-antimycotic and puromycin were purchased from Life Technologies (Grand Island, NY). Antibodies against c-KIT, PKC-A, phospho-PKC-A (Thr638), phospho-c-KIT (Tyr703), Tuberin (TSC2), phospho-ribosomal protein S6 kinase beta-1 (RPS6KB1, S6K1) at Thr389 site, phospho-Eukaryotic translation initiation factor 4E-binding protein 1 (EIF4EBP1, 4E-BP1) at Thr37/46 sites, Sequestosome-1 (SQSTM1, p62), microtubule-associated proteins 1A/1B light chain 3B (MAP1LC3B, LC3B), UBC9, TFAP2A, and autophagy protein 5 (ATG5) were purchased from Sigma-Aldrich (St Louis, MO).

### 2.2. Cell Culture

The human CRC HCT116 cells and LS174 cells were gifts from Laboratory of Molecular Biology, West China School of Basic Medical Sciences & Forensic Medicine, Sichuan University, China. HCT116 cells and LS174 cells were cultured in RPMI1640 medium supplemented with 10% BSA in PBS, 100 U/mL penicillin, 100 mg/ml streptomycin, and 2 mM glutamine and kept in a humidified atmosphere of 5% CO_2_ at 37°C.

### 2.3. Experimental Design

#### 2.3.1. Investigation of the Effective Concentration and Time for (+)-UA (<10 *μ*M) to Inhibit the Proliferation and Migration of HCT116 Cells and LS174 Cells

HCT116 cells and LS174 cells were divided into five groups: (1) 1% FBS medium, (2) 1% FBS medium + SCF (100 ng/ml), (3-5) 1% FBS medium + SCF (100 ng/ml) + (+)-UA (2, 4, or 8 *μ*M). After the treatments for 24 or 48 hours, Wound Healing Assay and Proliferation Assay were implemented, respectively. Before Migration Assay, cells were pretreated with 2, 4, or 8 *μ*M of (+)-UA for 24 or 48 hours, respectively. Migration Assay was carried out using the transwell plates. Cells were seeded on upper chamber, and chemotaxis medium containing 10% FBS with or without SCF was added to lower chamber. Cells were divided into eight groups: (1) 10% FBS medium + cells without pretreatment, (2) 10% FBS medium + SCF (100 ng/ml) + cells without pretreatment, and (3-8) 10% FBS medium + SCF (100 ng/ml) + the cells after pretreatments (2, 4, or 8 *μ*M of (+)-UA for 24 or 48 hours, respectively). After 12 hours of cells migration, migration indexes would be obtained. After that, the effective concentration and time for (+)-UA (<10 *μ*M) to inhibit the proliferation and migration of HCT116 cells and LS174 cells would be determined. The results indicated that the effective concentration was 8 *μ*M, and the effective time was 24 and 48 hours, respectively.

#### 2.3.2. Investigation of the Effects of (+)-UA (<10 *μ*M) on the Levels of ATP and LDH and Caspase-3/7 Activity in HCT116 Cells and LS174 Cells

Cells were pretreated with 2, 4, or 8 *μ*M of (+)-UA or DSMO for 6, 12, 24, or 48 hours, respectively. Cellular ATP level and LDH level were detected. Then, cells were pretreated with 2, 4, or 8 *μ*M of (+)-UA or DSMO for 24 or 48 hours, respectively. Caspase-3/7 (CASP3/7) activity measurements were implemented.

#### 2.3.3. Investigation of the Effects of 8 *μ*M (+)-UA on mTOR Pathway and Autophagy of HCT116 Cells

Phosphorylated TSC2 can inhibit mTOR activity [28]. Phosphorylation levels of S6K1 (Thr389) and 4E-BP1 (Thr37/46) could commendably represent mTOR activity [29]. LC3B-II has been widely used as a key marker of autophagosomes [30]. P62 acts as an autophagy-specific substrate, whose degradation level represents the increase extent of autophagic flux [30]. In the first experiment, HCT116 cells were pretreated with 8 *μ*M (+)-UA or DSMO for 24 or 48 hours, respectively. Since inhibition of TSC2 can induce the continuous activation of mTOR [28]. Silencing of TSC2 would be used to antagonize (+)-UA-mediated inhibition of mTOR, thereby verifying that (+)-UA-mediated autophagy is dependent on inhibition of mTOR. In the second experiment, HCT116 cells were divided into four groups: (1) scrambled siRNA (150 nM, 48 hours), (2) (+)-UA (8 *μ*M, 48 hours), (3) siRNA targeting TSC2 (150 nM, 48 hours), and (4) siRNA targeting TSC2 (150 nM, 48 hours) + (+)-UA (8 *μ*M, 48 hours). The levels of above-mentioned proteins were detected in these two experiments, respectively.

#### 2.3.4. Investigation of the Effects of 8 *μ*M of (+)-UA on c-KIT Expression and SCF-Mediated Activation of PKC-A and c-KIT in HCT116 Cells

Exogenous SCF was employed to induce phosphorylation of c-KIT (T703 site) and PKC-A (Thr638) [31]. Go-6976, an inhibitor of PKC [32], was employed to simulate PKC suppression. HCT116 cells were divided into seven groups: (1) DSMO as control, (2-3) (+)-UA (8 *μ*M, 24 or 48 hours), (4) SCF (100 ng/ml, 30 minutes), (5-6) (+)-UA (8 *μ*M, 24 or 48 hours) + SCF (100 ng/ml, 30 minutes), and (7) SCF (100 ng/ml, 30 min) + Go-6976 (6 *μ*M, 30 min). The levels of p-c-KIT, c-KIT, p-PKC-A, and p-PKC-A were detected, respectively. The results indicated that the treatment with 8 *μ*M (+)-UA mediated the downregulation of c-KIT and p-c-KIT in 48 hours and attenuated SCF-mediated activation of PKC-A in 24 or 48 hours, respectively.

#### 2.3.5. Investigation of the Effect of 8 *μ*M of (+)-UA on the Autophagic Degradation of c-KIT in HCT116 Cells

ATG5 is a key protein that is responsible for autophagosome formation [33]. Silencing of ATG5 was used to block (+)-UA-mediated autophagy. HCT116 cells were divided into six groups: (1) scrambled siRNA (150 nM, 48 hours), (2) siRNA targeting ATG5 (150 nM, 48 hours), (3) SCF (100 ng/ml, 30 minutes), (4) (+)-UA (8 *μ*M, 48 hours), (5) (+)-UA (8 *μ*M, 48 hours) + SCF (100 ng/ml, 30 minutes), and (6) siRNA targeting ATG5 (150 nM, 48 hours) + (+)-UA (8 *μ*M, 48 hours) + SCF (100 ng/ml, 30 minutes). The levels of c-KIT and p-c-KIT were detected, respectively.

#### 2.3.6. Investigation of the Effect of 8 *μ*M of (+)-UA on UBC9 Expression and the Transcriptional Inhibition of c-KIT in HCT116 Cells

In autophagy process, upregulated UBC9-mediated increase in sumoylation of TFAP2A (SUMO-TFAP2A) will result in transcriptional inhibition of c-KIT [11–13]. Silencing of UBC9 was employed to interfere UBC9-mediated SUMO-TFAP2A. HCT116 cells were divided into six groups: (1) scrambled siRNA (150 nM, 48 hours), (2) siRNA targeting UBC9 (150 nM, 48 hours), (3) SCF (100 ng/ml, 30 minutes), (4) (+)-UA (8 *μ*M, 48 hours), (5) (+)-UA (8 *μ*M, 48 hours) + SCF (100 ng/ml, 30 minutes), and (6) siRNA targeting UBC9 (150 nM, 48 hours) + (+)-UA (8 *μ*M, 48 hours) + SCF (100 ng/ml, 30 minutes). The protein levels of TFAP2A and SUMO-TFAP2A and the levels of protein and mRNA of UBC9 and c-KIT were detected, respectively.

### 2.4. Wound Healing Assay

HCT116 cells or LS174 cells were plated on 6-well culture plates at the density of 3 × 10^5^cells/well and were fed with 5% FBS. Until the cells grown to 95% confluence, monolayer cells were scratched to create a wound. The floating cells and 5% FBS medium were washed and removed. Then, the monolayer cells were fed with 1% FBS and treated with 2, 4, or 8 *μ*M of (+)-UA or DSMO as control for 24 or 48 h, respectively. Photographs were taken to detect the relative wound width under high power microscope. Experiments were repeated three times.

### 2.5. Migration Assay

After the pretreatments, 2 × 10^6^ cells/ml HCT116 cells or LS174 cells were seeded into the upper chamber of 24-well transwell plates and were starved in FBS-free medium for 2 h. After that, chemotaxis medium containing 10% FBS with or without SCF was added to lower chamber. Cells were incubated in FBS-free medium for 12 h at 37°C. Then, the cells on the upper chamber were removed. The cells in the lower chamber were stained. Photographs of cells migrated to the lower chamber were taken under an inverted microscope. The numbers of the cells were counted, and the data were expressed as Migration Index: compared to the number of cells in 10% FBS medium without SCF.

### 2.6. Proliferation Assay

HCT116 cells or LS174 cells were seeded into 96-well plates. After the pretreatments, proliferation assay was evaluated by BrdU Cell Proliferation Assay Kit (BioVision, USA) according to the manufacturer's protocol. Briefly, cells were treated with bromodeoxyuridine (BrdU, 10 *μ*L) and cultured at 37°C for 4 h. Then BrdU solution was washed and removed. FixDenat (200 *μ*l) was added. After incubation for 30 min, FixDenat was removed, and then anti-BrdU-POD (100 *μ*l) was added to experimental wells. After washing for three times, substrate solution (100 *μ*l) was added to experimental wells. And the optical density would be measured at 450 nm.

### 2.7. Cellular ATP Level Measurement

The ATP content was detected by the CellTiter-Glo® Luminescent Cell Viability Assay (Promega Corporation, WI, USA), according to the manufacturer's protocol.

### 2.8. Caspase-3/7 (CASP3/7) Activity Measurement

The activity of CASP3/7 was assayed by Caspase-Glo® 3/7 Assay Systems (Promega Corporation, WI, USA), according to the manufacturer's protocol.

### 2.9. LDH Assay

The cytotoxicity of (+)-UA was detected by Cytotoxicity LDH Assay Kit-WST® (Dojindo Molecular Technologies, Inc, US), according to the manufacturer's instructions.

### 2.10. Transient Transfection with siRNAs

The small interfering RNA (Stealth siRNA) for Human TSC2 (HSS111011), Human ATG5 (HSS190366), and Human UBC9 (HSS111130), and scrambled siRNA were from Thermo Fisher Scientific Inc. (MA, USA). HCT116 cells were seeded in 24-well plates (5 × 10^4^ cells per well) and allowed to grow overnight to reach 60%~70% confluence. siRNA was mixed with Lipofectamine RNAiMAX reagent (Thermo Fisher Scientific) and Opti-MEM medium (Thermo Fisher Scientific) and incubated at room temperature for 30 min. siRNA-reagent complex was added to cells and cultured for 48 hr. Individual experiments were repeated 3 times. Transient transfection of siRNAs was performed according to the manufacturer's protocol.

### 2.11. RT-qPCR

Total RNA was extracted from HCT116 cells using RNeasy Mini Kit (Qiagen), as recommended by the manufacturer's protocol. An on-column DNase digestion with the RNase-free DNase Set (Qiagen) was included to remove any genomic DNA contamination in RNA samples. RNA integrity was analyzed by agarose gel electrophoresis and its purity and concentration were calculated by measuring the optical density of the samples at 260 and 280 nm using a spectrophotometer. For single strand cDNA synthesis, 1 *μ*g of high quality purified RNA was reverse transcribed in a 20 *μ*L volume reaction using random hexamers and ThermoScript™ RT-PCR System, according to the manufacturer's instructions (Invitrogen). Prior to use on real-time PCR assays, the synthesized cDNA samples were diluted with nuclease-free water to a final concentration of 100 ng/*μ*L (roughly a 1:20 dilution). After that, cDNA was amplified using the SYBR Premix and following SYBR gene expression assays (Roche, USA). Primers were designed and shown as follows:

  c-KIT: 5′‐TATACAACCCTGGCATTATGTCC‐3′ (forward), 5′‐TGCGAAGGAGGCTAAACCTA‐3′ (reverse);
  UBC9: 5′‐TTATGAACTGGGAATGTGC‐3′ (forward),
  5′‐CTTTGGAGGTGATGAGGG‐3′ (reverse).

The relative amount of mRNA to *β*-Actin RNA was described using the equation 2^−ΔCT^ where ^Δ^CT=CT of mRNA-CT of *β*-Actin [34].

### 2.12. Western Blot Analysis

Total protein was extracted from the HCT116 cells, and the cells were washed twice with PBS and resuspended with an ice-cold Tris-mannitol buffer (2 mM Tris, 50 mM Mannitol, pH 7.1) containing protease inhibitors and 0.02% sodium azide. Samples were centrifuged for 10 minutes at 950 g. After the concentrations of the proteins were determined, equal amounts of protein were electrophoresed on sodium dodecyl sulfate-polyacrylamide gel and transferred to polyvinylidene difluoride membranes (Sigma-Aldrich, MO, USA). The membranes were blocked with 5% non-fat dry milk in Tis-buffer saline-0.05% Tween (pH 7.4) and incubated with target antibodies (Abcam) at 4°C overnight, followed by incubation with horseradish peroxidase (HRP)-conjugated secondary antibodies at 37°C for 2 h. The signals of targeted protein were tested by Amersham ECL (Sigma-Aldrich, MO, USA). And glyceraldehydes-3-phosphate dehydrogenase (GAPDH, Boster) was used as an internal control to correct variation among the different samples.

### 2.13. Statistical Analysis

Data were indicated as mean ± standard deviation (SD). The data were analyzed by one-way ANOVA followed by the least significant difference (LSD) test using SPSS 19.0 software. Statistical difference was accepted at *P* < 0.05.

## 3. Results

### 3.1. 8 *μ*M of (+)-UA Inhibited SCF-Mediated Proliferation and Migration of CRC c-KIT(+) Cells

**Figure 1:**
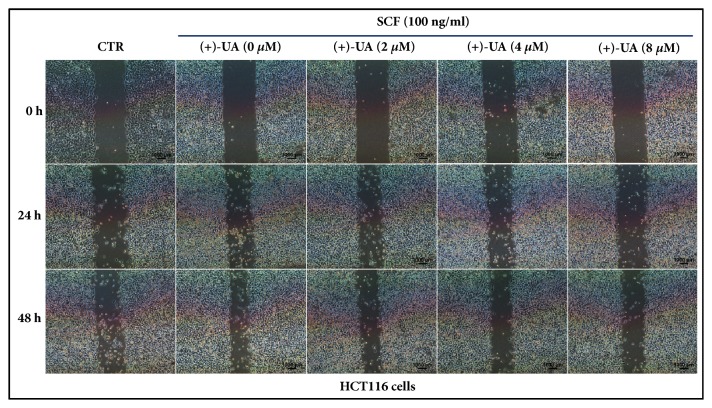
Visualization of the inhibiting effects of (+)-UA on SCF-mediated wound closure of HCT116 cells.

**Figure 2:**
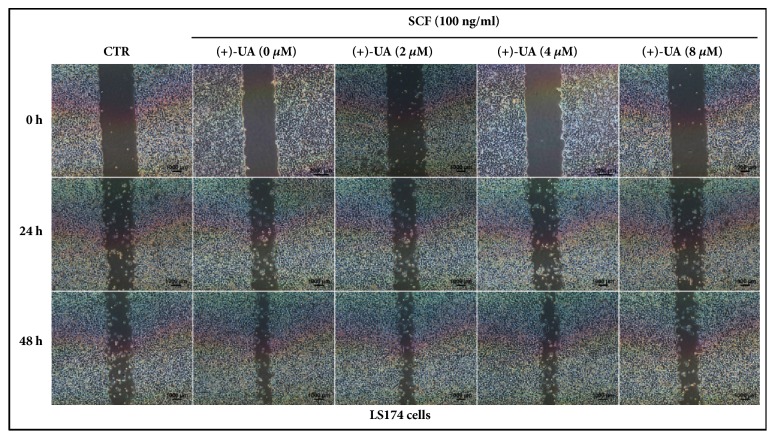
Visualization of the inhibiting effects of (+)-UA on SCF-mediated wound closure of LS174 cells.

**Figure 3:**
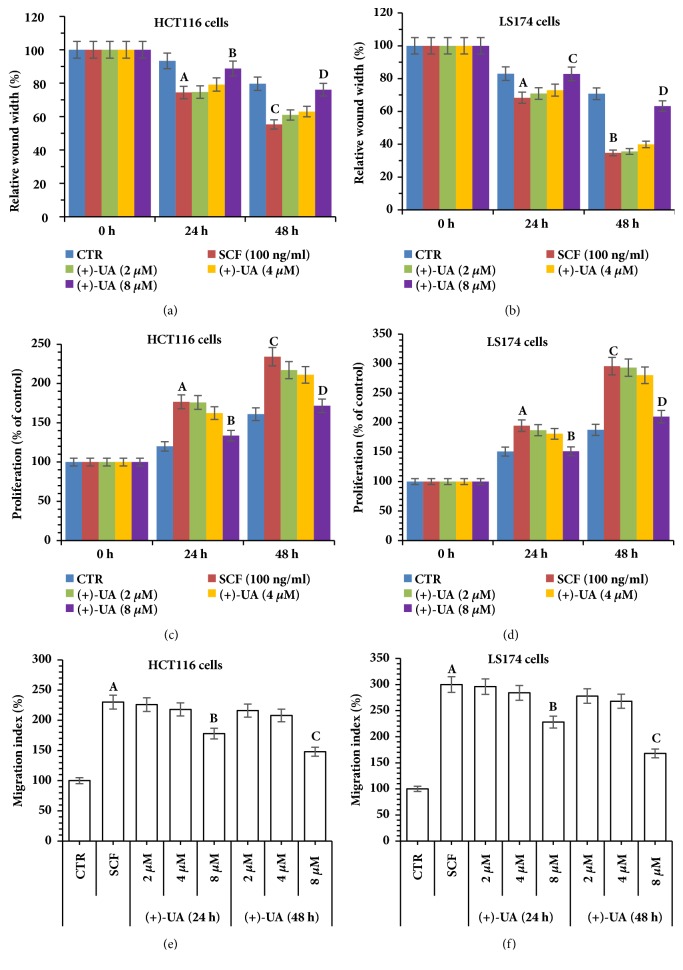
**8** ***μ*** **M of (+)-UA inhibited SCF-mediated proliferation and migration of CRC c-KIT(+) cells. (a-b)** Wound Healing Assay: ^a^*P* < 0.05, versus CTR (24 h); ^b^*P* < 0.05, versus SCF (24 h); ^c^*P* < 0.05, versus CTR (48 h); ^d^*P* < 0.05, versus SCF (48 h).** (c-d) Cell proliferation assay**: ^a^*P* < 0.05, versus CTR (24 h); ^b^*P* < 0.05, versus SCF (24 h); ^c^*P* < 0.05, versus CTR (48 h); ^d^*P* < 0.05, versus SCF (48 h).** (e-f) Migration Assay**: ^a^*P* < 0.01, versus CTR; ^b^*P* < 0.05, versus SCF; ^c^*P* < 0.05, versus SCF.

In the wound healing assays, the inhibiting effects of (+)-UA on SCF-mediated wound closure of HCT116 cells and LS174 cells were visualized in Figures 1 and 2, respectively. Further studies indicated that the treatments of HCT116 cells and LS174 cells with 8 *μ*M of (+)-UA for 24 or 48 hours evidently inhibited SCF-mediated increases in the proliferation and migration of cells (Figures 3(c)–3(f)). Consistent with these results, the treatments of HCT116 cells and LS174 cells with 8 *μ*M of (+)-UA for 24 or 48 hours demonstrated the significant inhibiting effects on SCF-mediated wound closure (Figures 3(a) and 3(b)).

### 3.2. 8 *μ*M of (+)-UA Decreased Cellular ATP Content and Increased LDH Release

**Figure 4:**
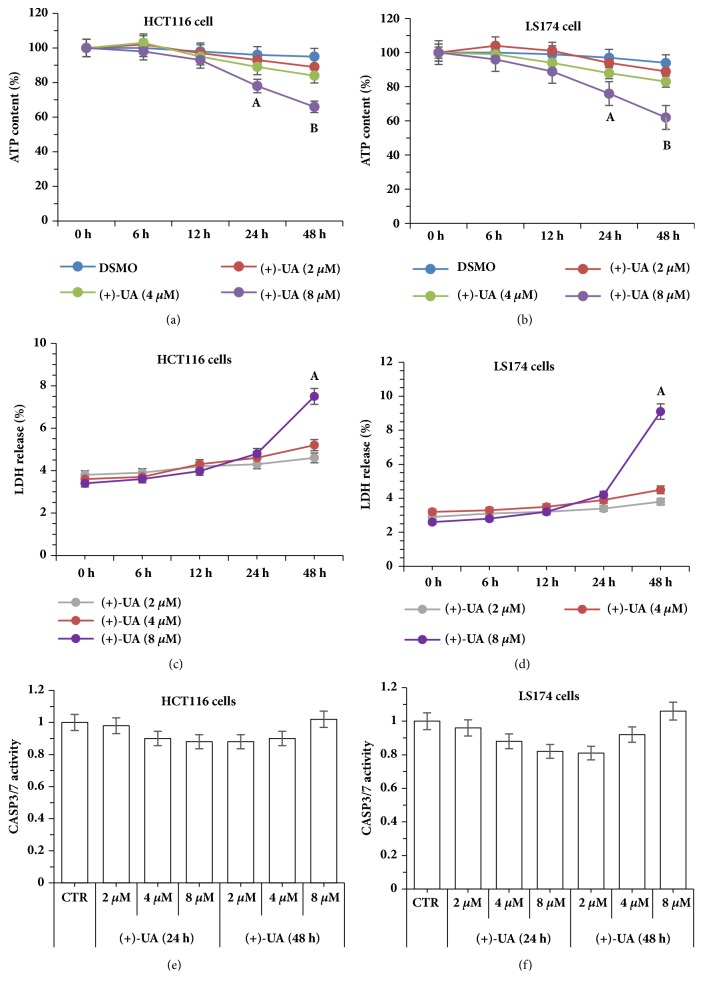
**8** ***μ*** **M of (+)-UA decreased cellular ATP content and increased LDH release. (a-b) **Cellular ATP level measurement: ^a^*P* < 0.05, and ^b^*P* < 0.05, versus control (0 h);** (c-d)** LDH level measurement: ^a^*P* < 0.05, versus control (0 h);** (e-f)** CASP3/7 activity assay: 2-8 *μ*M of (+)-UA did not significantly induce increase in CASP3/7 activity.

Firstly, the treatment of HCT116 cells or LS174 cells with 8 *μ*M of (+)-UA for 24 or 48 hours significantly decreased cellular ATP content (Figures 4(a) and 4(b)), which suggested an uncoupling effect. Secondly, the treatment of HCT116 cells or LS174 cells with 8 *μ*M of (+)-UA for 48 hours evidently increased LDH release (Figures 4(c) and 4(d)). Notably, the treatments of HCT116 cells or LS174 cells with 2-8 *μ*M of (+)-UA did not significantly induce increase in CASP3/7 activity (Figures 4(e) and 4(f)), which suggested the possible absence of induction of apoptosis.

### 3.3. 8 *μ*M of (+)-UA Induced Autophagy via Inhibition of mTOR

**Figure 5:**
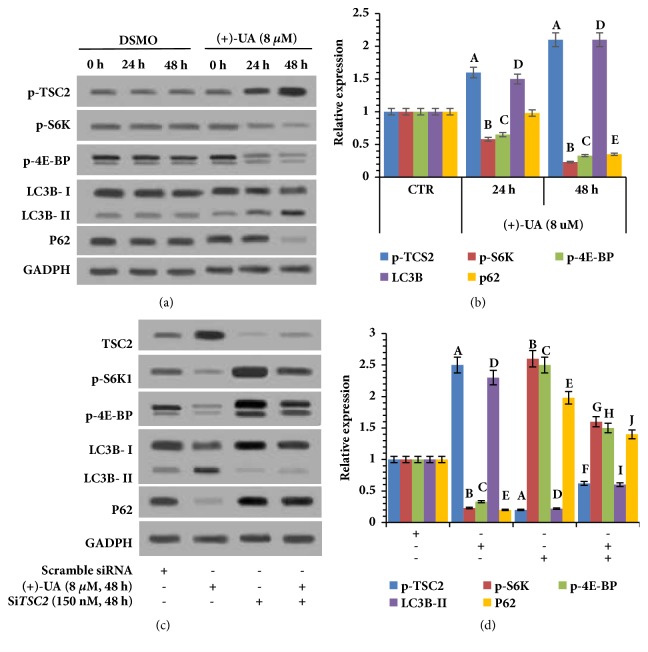
**8** ***μ*** **M of (+)-UA induced autophagy via inhibition of mTOR. (a) **Western blot: the treatment of HCT116 cells with 8 *μ*M of (+)-UA mediated an autophagy with a complete autophagic flux in 48 hours rather than in 24 hours.** (b) **Quantitative analysis: ^a^*P* < 0.05, ^b^*P* < 0.05, ^c^*P* < 0.05, ^d^*P* < 0.05, and ^e^*P* < 0.05, versus CTR.** (c) **Western blot: (+)-UA-mediated autophagy was dependent on inhibition of mTOR.** (d) **Quantitative analysis: ^a^*P* < 0.05, ^b^*P* < 0.05, ^c^*P* < 0.05, ^d^*P* < 0.05, and ^e^*P* < 0.05, versus scramble siRNA, ^f^*P* < 0.05, ^g^*P* < 0.05, ^h^*P* < 0.05, ^i^*P* < 0.05, and ^j^*P* < 0.05, versus (+)-UA.

Western blot revealed that the treatment of HCT116 cells with 8 *μ*M of (+)-UA for 24 or 48 hours markedly upregulated phosphorylation level of TSC2 and downregulated the phosphorylation levels of S6K1 and 4E-BP1 and induced a significant transformation of LC3B from cytosolic form (LC3B-I) to membrane-bound form (LC3B-II) (Figures 5(a) and 5(b)), which suggested the formation of autolysosomes. Notably, the degradation of P62 did not take place under the treatment of HCT116 cells with 8 *μ*M (+)-UA for 24 hours, but that occurred under the treatment with 8 *μ*M (+)-UA for 48 hours (Figures 5(a) and 5(b)). More importantly, silencing of TSC2 significantly attenuated (+)-UA-mediated upregulation of p-TSC2 and the downregulation of p-S6K1 and p-4E-BP1 and inhibited (+)-UA-mediated LC3B-II upregulation and P62 degradation (Figures 5(c) and 5(d)).

### 3.4. 8 *μ*M (+)-UA Modulated c-KIT Expression and SCF-Mediated Activation of PKC-A and c-KIT

**Figure 6:**
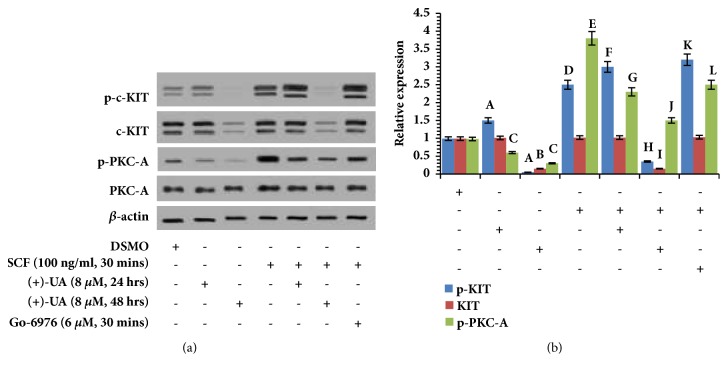
**8** ***μ*** **M (+)-UA modulated c-KIT expression and SCF-mediated activation of PKC-A and c-KIT. (a) **Western blot: (1) the treatment of HCT116 cells with 8 *μ*M (+)-UA for 24 and 48 hours attenuated SCF-induced upregulation of p-PKC-A; (2) the treatment of HCT116 cells with 8 *μ*M (+)-UA for 48 hours not only reversed SCF-induced upregulation of p-c-KIT but also mediated downregulation of c-KIT.** (b) **Quantitative analysis: ^a^*P* < 0.05, ^b^*P* < 0.05, ^c^*P* < 0.05, ^d^*P* < 0.05, and ^e^*P* < 0.05, versus DSMO; ^f^*P* < 0.05, ^g^*P* < 0.05, ^h^*P* < 0.05, ^i^*P* < 0.05, ^j^*P* < 0.05, ^k^*P* < 0.05, and ^l^*P* < 0.05, versus SCF.

Western blot (Figures 6(a) and 6(b)) revealed that (1) the treatment of HCT116 cells with 8 *μ*M (+)-UA for 24 hours mediated the significant downregulation of p-PKC-A and the distinct upregulation of p-c-KIT; (2) the treatment of HCT116 cells with 8 *μ*M (+)-UA for 48 hours induced the notable downregulation of p-PKC-A, p-c-KIT, and c-KIT; (3) the treatment of HCT116 cells with SCF for 30 minutes mediated the significant upregulation of p-c-KIT and p-PKC-A; (4) the treatment of HCT116 cells with 8 *μ*M (+)-UA for 24 hours evidently attenuated SCF-induced upregulation of p-PKC-A but further enhanced SCF-induced upregulation of p-c-KIT; (5) the treatment of HCT116 cells with 8 *μ*M (+)-UA for 48 hours not only reversed SCF-induced upregulation of p-c-KIT but also mediated a notable downregulation of c-KIT; (6) the effects of Go-6976 on p-PKC-A and p-c-KIT showed the same trend as that of the treatment with (+)-UA for 24 hours.

### 3.5. 8 *μ*M of (+)-UA Induced Autophagic Degradation of c-KIT

**Figure 7:**
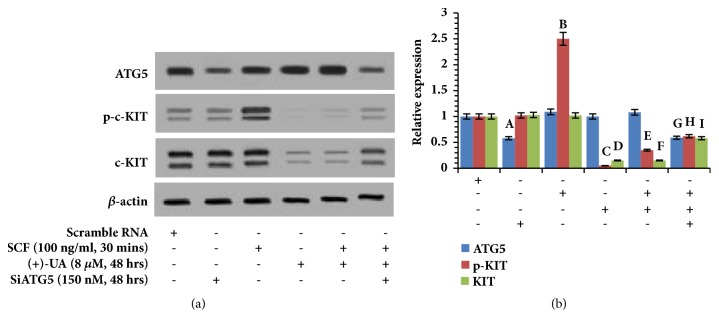
**8** ***μ*** **M of (+)-UA induced autophagic degradation of c-KIT. (a) **Western blot: the treatment of HCT116 cells with 8 *μ*M of (+)-UA for 48 h not only attenuated SCF-induced upregulation of p-c-KIT but also downregulated c-KIT, which were rescued by silencing of ATG5.** (b) **Quantitative analysis: ^a^*P* < 0.05, ^b^*P* < 0.05, ^c^*P* < 0.05, and ^d^*P* < 0.05, versus scramble RNA; ^e^*P* < 0.05, and ^f^*P* < 0.05, versus SCF; ^g^*P* < 0.05, ^h^*P* < 0.05, and ^i^*P* < 0.05, versus SCF + (+)-UA.

Western blot (Figures 7(a) and 7(b)) revealed that (1) the treatment of HCT116 cells with SCF for 30 minutes significantly upregulated the expression of p-c-KIT; (2) the treatment of HCT116 cells with 8 *μ*M of (+)-UA for 48 hours not only reversed SCF-induced upregulation of p-c-KIT but also mediated a notable downregulation of c-KIT; (3) the downregulation of p-c-KIT and c-KIT, induced by (+)-UA, was rescued by silencing of ATG5.

### 3.6. 8 *μ*M of (+)-UA Induced Transcriptional Inhibition of c-KIT

**Figure 8:**
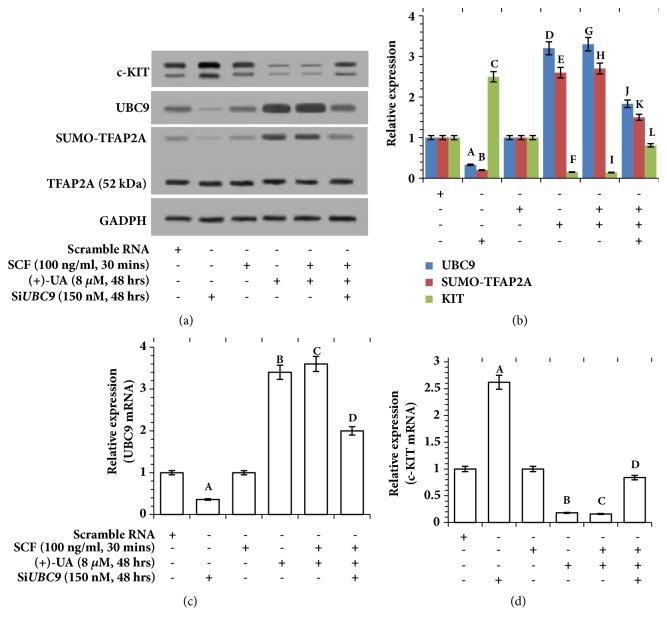
**8** ***μ*** **M of (+)-UA induced transcriptional inhibition of c-KIT. (a) **Western blot: the treatments of HCT116 cells with (+)-UA (8 *μ*M, 48 hours) or (+)-UA (8 *μ*M, 48 hours) + SCF (100 ng/ml, 30 minutes) induced UBC9 upregulation, thereby mediating the increase in SUMO-TFAP2A, which ultimately resulted in the downregulation of c-KIT, which were reversed by silencing UBC9.** (b) **Quantitative analysis: ^a^*P* < 0.05, ^b^*P* < 0.05, ^c^*P* < 0.05, ^d^*P* < 0.05, ^e^*P* < 0.05, ^f^*P* < 0.05, ^g^*P* < 0.05, ^h^*P* < 0.05, and ^i^*P* < 0.05, versus scramble RNA; ^j^*P* < 0.05, ^k^*P* < 0.05, and ^l^*P* < 0.05, versus SCF + (+)-UA. (**c-d) **RT-qPCR: ^a^*P* < 0.05, ^b^*P* < 0.05, and ^c^*P* < 0.05, versus scrambled siRNA; ^d^*P* < 0.05, versus SCF + (+)-UA.

Western blot (Figures 8(a) and 8(b)) revealed that (1) the treatment of HCT116 cells with SCF for 30 minutes did not induce the expression changes of UBC9, SUMO-TFAP2A, TFAP2A, and c-KIT; (2) the treatments of HCT116 cells with (+)-UA (8 *μ*M, 48 hours) or (+)-UA (8 *μ*M, 48 hours) + SCF (100 ng/ml, 30 minutes) evidently induced upregulation of UBC9, thereby mediating the increase in the upper band of TFAP2A (SUMO-TFAP2A), which ultimately resulted in downregulation of c-KIT; (3) the expression changes of UBC9 and SUMO-TFAP2A as well as c-KIT, induced by the treatment with (+)-UA + SCF, were reversed by silencing UBC9. Consistent with the results in Figures 8(a) and 8(b), RT-qPCR verified that (1) the treatments of HCT116 cells with (+)-UA (8 *μ*M, 48 hours) or (+)-UA (8 *μ*M, 48 hours) + SCF (100 ng/ml, 30 minutes) mediated a significant upregulation of UBC9 mRNA, which were remarkably attenuated by silencing UBC9 (Figure 8(c)); (2) the treatments of HCT116 cells with (+)-UA (8 *μ*M, 48 hours) or (+)-UA (8 *μ*M, 48 hours) + SCF (100 ng/ml, 30 minutes) induced an outstanding downregulation of c-KIT mRNA, which were significantly rescued by silencing UBC9 (Figure 8(d)).

## 4. Discussion

Inhibition of tumor cells migration is a therapeutic strategy for CRC patients [3]. SCF-dependent activation of c-KIT is responsible for migration of c-KIT(+) CRC cells [6]. However, drug resistance to Imatinib Mesylate (a c-KIT inhibitor) has emerged [9]. Inhibition of mTOR can induce autophagic degradation of c-KIT [10]. As a novel mTOR inhibitor, (+)-UA, isolated from lichens, has two major pharmacological functions including targeting inhibition of mTOR and induction of proton shuttle [18, 19]. To reduce the adverse reaction of liver injury, the treatment concentration of (+)-UA on cells should be limited to lower than 10 *μ*M [24]. This study has proved that (+)-UA (<10 *μ*M) could effectively inhibit migration of c-KIT(+) CRC cells.

*(+)-UA Inhibited SCF-Mediated Migration of c-KIT(+) CRC Cells*. c-KIT is expressed in HCT116 cell line and LS174 cell line, respectively [5, 27]. The treatments of HCT116 cells or LS174 cells with 8 *μ*M of (+)-UA for 24 or 48 hours could effectively inhibit SCF-mediated cell proliferation (Figures 3(c) and 3(d)), which contribute to delay wound closure in Wound Healing Assay (Figures 1, 2, 3(a), and 3(b)). More importantly, the Migration Assay confirmed that the treatments of HCT116 cells or LS174 cells with 8 *μ*M of (+)-UA for 24 or 48 hours significantly inhibited SCF-mediated cell migration (Figures 3(e) and 3(f)). The evidences suggested that 8 *μ*M could be determined as the effective concentration for (+)-UA to inhibit SCF-mediated migration of CRC c-KIT(+) cells.

*(+)-UA Induced ATP Decrease via Uncoupling.* Lipophilic- and weakly acidic- (+)-UA would mediate mitochondrial proton shuttle (uncoupling) to induce ATP decrease [19]. ATP decrease would directly inhibit cell motility [20]. This study verified that the treatment of HCT116 cells or LS174 cells with 8 *μ*M of (+)-UA for 24 or 48 hours observably decreased ATP levels (Figures 4(a) and 4(b)), thereby remarkably inhibiting cell migration (Figures 3(e) and 3(f)). These results suggested that the treatment of HCT116 cells and LS174 cells with 8 *μ*M of (+)-UA could mediate inhibition of cells migration probably via uncoupling-induced ATP decrease.

*(+)-UA Induced Inhibition of mTORC1 through the Functional Synergy between Uncoupling and the Targeting Inhibition of mTOR. *Firstly, (+)-UA could mediate suppression of mTOR via the target-binding of mTOR [18]. Secondly, uncoupling-induced ATP decrease would mediate the activation of 5′-AMP-activated protein kinase, catalytic alpha subunit (AMPK), thereby inducing the increase in phosphorylation level of TSC2, which ultimately resulted in inhibition of mTORC1 [19, 28]. Therefore, (+)-UA-mediated uncoupling and the targeting inhibition of mTOR synergistically mediated the inhibition of mTOR. As the results of uncoupling-induced ATP decrease and the targeting inhibition of mTOR, treatment of HCT116 cells with 8 *μ*M of (+)-UA for 24 or 48 h evidently upregulated TSC2 and downregulated the phosphorylation levels of S6K1 and 4E-BP1 (Figures 5(a) and 5(b)). More importantly, silencing of TSC2 significantly attenuated (+)-UA-mediated upregulation of TSC2 and also downregulation of p-S6K1 and p-4E-BP1 and inhibited (+)-UA-mediated LC3B-II upregulation and P62 degradation (Figures 5(c) and 5(d)). These evidences suggested that (1) (+)-UA-mediated inhibition of mTOR is partially dependent on uncoupling-mediated ATP decrease and TSC2 activation and (2) (+)-UA-mediated autophagy is dependent on inhibition of mTOR. Furthermore, phosphorylation of S6K1 will promote cell migration not only via increasing MMP-9 expression and the phosphorylation level of focal adhesion proteins but also via inducing F-actin reorganization [35, 36]. Phosphorylation of 4E-BP1 at multiple site will also promote F-actin reorganization [36]. Consequently, (+)-UA-mediated inhibition of cells migration may be partially dependent on mTORC1 inhibition that was achieved through the functional synergy between the targeting inhibition of mTOR and uncoupling.

*(+)-UA-Induced LDH Release Was Dependent on the Functional Synergy between Targeting Inhibition of mTOR and the Inductions of Proton Shuttle in Lysosomes and Autophagosome*. (+)-UA could mediate inhibition of mTOR [18]. Meanwhile, (+)-UA would also mediate proton shuttle in lysosomes and autophagosome and result in autophagosome dysmaturity and damage to lysosome acidification [19]. This study showed that the degradation of P62 did not take place under the treatment of HCT116 cells with 8 *μ*M (+)-UA for 24 hours, but that occurred under the treatment with 8 *μ*M (+)-UA for 48 hours (Figures 5(a) and 5(b)), which indicated the conversion from an incomplete autophagic flux to a complete autophagic flux. But (+)-UA-mediated proton shuttle in lysosomes and autophagosome would result in cytoplasm acidification [19]. Therefore, treatment of HCT116 cells and LS174 cells with 8 *μ*M of (+)-UA for 48 hours evidently induced LDH release (Figures 4(c) and 4(d)), which suggested the potential presence of necrosis death. Moreover, the treatment of HCT116 cells and LS174 cells with 8 *μ*M of (+)-UA for 24 or 48 hours did not remarkably induce the increase in CASP3/7 activity (Figures 4(e) and 4(f)), which suggested that treatment of some cancer cell lines with low dose of (+)-UA is not yet sufficient to increase the permeability of mitochondrial membrane [19]. These evidences indicated that the treatment of HCT116 cells with 8 *μ*M of (+)-UA for 24 or 48 h may not induce apoptosis, but probably mediate necrosis death. Therefore, the treatment of HCT116 cells with 8 *μ*M of (+)-UA-mediated inhibition of cells migration should also be partially attributed to necrosis death pathway that was initiated by the functional synergy between targeting inhibition of mTOR and the inductions of proton shuttle in lysosomes and autophagosome.

*(+)-UA Induced Activity Suppression of PKC-A*. Treatment of HCT116 cells with 8 *μ*M (+)-UA within the early 24 or 48 hours evidently reversed SCF-induced upregulation of p-PKC-A (Figures 6(a) and 6(b)), which suggested the activity suppression of PKC-A mediated by mTORC2 inhibition [14]. In the SCF stimulation-mediated feedback loop, PKC-A can induce phosphorylation of c-KIT on serine residues, which will lead to inhibition of tyrosine autophosphorylation of c-KIT [37]. This study verified the feedback loop that 8 *μ*M (+)-UA-mediated PKC-A activity inhibition in 24 hours further enhanced SCF-induced tyrosine autophosphorylation of c-KIT (Figures 6(a) and 6(b)). Crucially, PKC-A activity was necessary for SCF-mediated cells motility [37]. This study proved that treatment of HCT116 cells with 8 *μ*M (+)-UA for 24 or 48 hours remarkably reversed SCF-mediated cells migration (Figures 3(e) and 3(f)). Hence, treatment of HCT116 cells with 8 *μ*M (+)-UA in 24 or 48 hours could inhibit SCF-mediated cells migration probably via activity suppression of PKC-A.

*Autophagic Degradation of c-KIT*. In 24 h, 8 *μ*M of (+)-UA-mediated proton shuttle in lysosomes and autophagosome resulted in the autophagy only with the incomplete autophagic flux (Figures 5(a) and 5(b)), which were insufficient to mediate degradation of c-KIT (Figures 6(a) and 6(b)). With prolonged exposure time, an autophagy of a complete autophagic flux had been successfully induced in 48 h (Figures 5(a) and 5(b)). Sequestosome-1 (SQSTM1) and histone deacetylase 6 (HDAC6) would probably contribute to selective autophagic degradation of c-KIT [10]. This study found that the treatment of HCT116 cells with 8 *μ*M of (+)-UA for 48 h not only attenuated SCF-induced upregulation of p-c-KIT but also downregulated c-KIT, which were rescued by silencing of ATG5 (Figures 7(a) and 7(b)). These evidences confirmed that the treatment of HCT116 cells with 8 *μ*M of (+)-UA for 48 h effectively induced autophagic degradation of c-KIT.

*Transcriptional Inhibition of c-KIT in Autophagy Process*. The upregulation of UBC9 is required for increasing autophagic flux in autophagy process [11]. However, UBC9 will also be degraded by lysosomes and autophagosome in the increasing autophagic flux [38]. This study found that treatment of HCT116 cells with 8 *μ*M of (+)-UA for 48 h induced the significant upregulation of mRNA and protein of UBC9 (Figures 8(a), 8(b), and 8(c)). This evidence suggested that the treatment of HCT116 cells with 8 *μ*M of (+)-UA for 48 hours mediated a balance between transcription and degradation of UBC9 that maintains upregulated level of UBC9. UBC9 interacts with the C terminal region of TFAP2A, which lead to sumoylation of TFAP2A on lysine-10 [12]. Therefore, 8 *μ*M (+)-UA-mediated UBC9 upregulation effectively induced the increase in SUMO-TFAP2A (Figures 8(a) and 8(b)). After that, sumoylation of TFAP2A will inhibit its ability to binding TFAP2A dimer to the KIT gene promoter that is required for stimulating KIT transcription [13]. RT-qPCR (Figure 8(d)) and Western blot (Figures 8(a) and 8(b)) verified that treatment of HCT116 cells with 8 *μ*M (+)-UA for 48 hours induced the notable downregulation of mRNA and protein of c-KIT, which were significantly rescued by silencing of UBC9. These data suggested that treatment of HCT116 cells with 8 *μ*M of (+)-UA for 48 h induced transcriptional inhibition of c-KIT via upregulated UBC9-mediated suppression of AP-2 transcriptional activity.

Repression of c-KIT is closely associated with reduced migration of c-KIT(+) cells [7]. Therefore, (+)-UA-mediated inhibition of SCF-mediated cells migration would probably be mainly dependent on the autophagic degradation and transcriptional inhibition of c-KIT. Taken together, (+)-UA at 8 *μ*M, a relative low hepatocyte toxicity concentration, could be determined as the effective concentration to inhibit SCF-mediated migration of c-KIT(+) CRC cells. (+)-UA-mediated functional synergy between induction of proton shuttle and targeting inhibition of mTOR effectively regulated many processes related to cell migration, thereby inhibiting SCF-mediated migration of c-KIT(+) CRC cells.

## Data Availability

The data used to support the findings of this study are available from the corresponding author upon request.
